# Detecting visually significant cataract using retinal photograph-based deep learning

**DOI:** 10.1038/s43587-022-00171-6

**Published:** 2022-02-21

**Authors:** Yih-Chung Tham, Jocelyn Hui Lin Goh, Ayesha Anees, Xiaofeng Lei, Tyler Hyungtaek Rim, Miao-Li Chee, Ya Xing Wang, Jost B. Jonas, Sahil Thakur, Zhen Ling Teo, Ning Cheung, Haslina Hamzah, Gavin S. W. Tan, Rahat Husain, Charumathi Sabanayagam, Jie Jin Wang, Qingyu Chen, Zhiyong Lu, Tiarnan D. Keenan, Emily Y. Chew, Ava Grace Tan, Paul Mitchell, Rick S. M. Goh, Xinxing Xu, Yong Liu, Tien Yin Wong, Ching-Yu Cheng

**Affiliations:** 1 Singapore Eye Research Institute, Singapore National Eye Centre, Singapore, Singapore; 2 Duke-NUS Medical School, Singapore, Singapore; 3 Department of Ophthalmology, Yong Loo Lin School of Medicine, National University of Singapore, Singapore, Singapore; 4 Institute of High Performance Computing, A*STAR, Singapore, Singapore; 5 Beijing Institute of Ophthalmology, Beijing Ophthalmology and Visual Science Key Lab, Beijing, China; 6 Department of Ophthalmology, Medical Faculty Mannheim of the Ruprecht-Karis-University Heidelberg, Mannheim, Germany; 7 National Center for Biotechnology Information, National Library of Medicine, National Institutes of Health, Bethesda, MD USA; 8 National Eye Institute, National Institutes of Health, Bethesda, MD USA; 9 Centre for Vision Research, Department of Ophthalmology, The Westmead Institute for Medical Research, University of Sydney, Westmead Hospital, Westmead, New South Wales Australia; 10 National Health Medical Research Council Clinical Trials Centre, University of Sydney, Sydney, New South Wales Australia; 11 Department of Ophthalmology, Yong Loo Lin School of Medicine, National University of Singapore, Singapore, Singapore

**Keywords:** Diagnostic markers, Predictive markers, Ageing

## Abstract

Age-related cataracts are the leading cause of visual impairment among older adults. Many significant cases remain undiagnosed or neglected in communities, due to limited availability or accessibility to cataract screening. In the present study, we report the development and validation of a retinal photograph-based, deep-learning algorithm for automated detection of visually significant cataracts, using more than 25,000 images from population-based studies. In the internal test set, the area under the receiver operating characteristic curve (AUROC) was 96.6%. External testing performed across three studies showed AUROCs of 91.6–96.5%. In a separate test set of 186 eyes, we further compared the algorithm’s performance with 4 ophthalmologists’ evaluations. The algorithm performed comparably, if not being slightly more superior (sensitivity of 93.3% versus 51.7–96.6% by ophthalmologists and specificity of 99.0% versus 90.7–97.9% by ophthalmologists). Our findings show the potential of a retinal photograph-based screening tool for visually significant cataracts among older adults, providing more appropriate referrals to tertiary eye centers.

## Abstract (web summary)

Age-related cataracts are characterized by clouding of the eye’s lens and cause vision impairment or loss. Here the authors develop a retinal photograph-based deep-leaning method to detect visually significant cataracts and report that it detects cataracts with similar accuracy to ophthalmologists.

## Main

Age-related cataracts are the leading cause of disease-related visual impairment globally, accounting for 94 million adults aged ≥50 years who experienced low vision or blindness in 2020 (ref. ^1^). Although a cataract is easily treatable, a significant number of patients with a visually significant cataract (that is, a cataract with severe visual loss) remain undiagnosed in communities, especially in rural areas, due to the limited availability of, or accessibility to, cataract screening^2,3^. Based on a previous report in an Asian population, up to 68.8% of older adults with visually significant cataracts were not aware of having the condition^2^. Hence, there is a critical need to facilitate access to cataract screening for earlier surgical intervention. This is also important given that cataract surgery is a highly cost-effective intervention^4,5^.

The conventional approach for cataract diagnosis relies mainly on the assessment of the human crystalline lens using slit-lamp biomicroscopy, operated by trained ophthalmologists. However, this conventional approach poses a major challenge in lower-income countries or rural communities where there are shortages of trained ophthalmologists^6^. In other high-income countries, although community eye-screening programs, such as a diabetic retinopathy (DR)-screening program, are in place, they generally do not include slit-lamp-based examinations or have ophthalmologists on site to examine for cataracts. Hence, the traditional ophthalmologist-dependent model has limited reach and screening capacity, if applied to community screening. An automated, deep-learning algorithm that can detect visually significant cataracts based on retinal photographs may help to address this issue. The development of such a system has remained relatively unexplored^7^.

Although some previous studies reported retinal photograph-based, deep-learning algorithms for detecting cataracts, these algorithms had focused only on the presence of cataracts, without considering the vision status^8–12^. Such algorithms would probably result in over-referrals of mild/nonvision-threatening cataract cases, who might not require surgery for many years. These earlier studies also did not demonstrate the algorithms’ performance in external validations. Moreover, these previous studies were flawed in their ground truth establishment, in which cataract grading was determined in a nonstandardized way, based solely on subjective judgment of ‘haziness level’ on a retinal photograph.

To address these gaps, using a total of 25,742 retinal photographs, we designed and tested a new retinal photograph-based, deep-learning algorithm for identification of visually significant cataracts. Such an algorithm would potentially serve as a more efficient cataract-screening tool in the community. Furthermore, given the increasing availability of retinal cameras and their increasing use in community eye-screening programs, this new algorithm could be potentially adopted and integrated into existing screening programs.

## Results

We developed the deep-learning algorithm using retinal fundus images of 4,138 study participants (8,045 eyes) as a development set from the Singapore Malay Eye Study (SIMES) cohort study. We validated the performance of the algorithms using retinal images from 900 individuals (1,692 eyes) as an internal test set from the SIMES cohort, and then tested this further using 3 external test sets including 8,444 individuals (16,005 eyes) from the Singapore Chinese Eye Study (SCES), Singapore Indian Eye Study (SINDI) and Beijing Eye Study (BES). The mean (±s.d.) of age was 59.4 (±10.2) years in the internal test set of the SIMES cohort. Although the mean age of the participants in the external test sets ranged from 57.1 ± 9.1 years in SINDI to 63.9 ± 9.3 years in BES, across all the included datasets, the prevalence of visually significant cataracts (by eyes) ranged from 1.04% to 6.05%. The demographics and characteristics of the study participants are summarized in Table 1.

**Table 1:** Characteristics of development and testing datasets

Characteristics	Development set	Internal test set	External test sets		
	SIMES	SIMES	SCES	SINDI	BES
Number of patients	4,138	900	3,011	2,945	2,488
Number of eyes	8,045	1,692	5,747	5,626	4,632
Age, years (s.d.)	60.8 (11.0)	59.41 (10.21)	58.43 (9.22)	57.13 (9.07)	63.87 (9.30)
Male gender, no. (%)	1,951 (47.2)	430 (47.78)	1,638 (49.82)	1,498 (50.87)	1,060 (42.60)
Visually significant cataract^a^ (by eye), no. (%)	487 (6.1)	72 (4.26)	141 (2.45)	138 (2.45)	48 (1.04)

Data presented as mean (s.d.) or no. (percentage), where appropriate.

^a^Cataract with BCVA < 20/60.

**Fig. 1:**
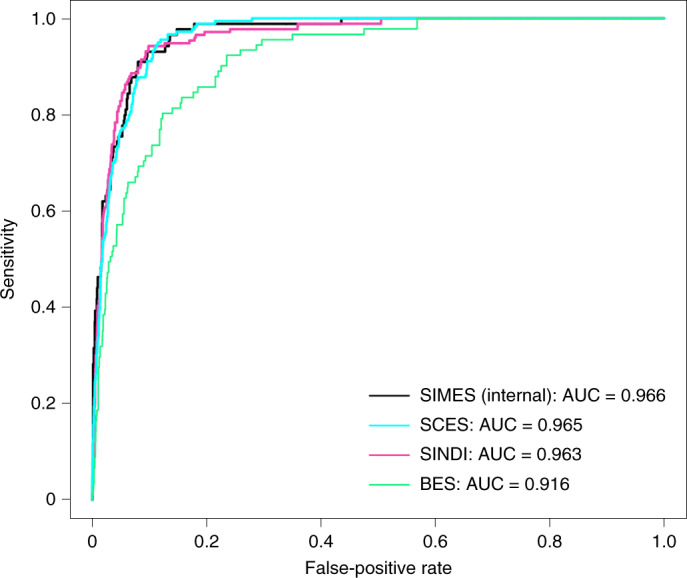
ROC curve showing performance of the classification algorithm for the detection of visually significant cataracts (defined as BCVA < 20/60).

We first examined the performance of the deep-learning-based, classification algorithm in detecting visually significant cataracts. In the internal test set, the algorithm’s AUROC for detecting visually significant cataracts was 96.6% (95% confidence interval (CI) 95.5–97.7), with a sensitivity of 95.7% and a specificity of 89.0%. Across the three external tests, the AUROC for detection of visually significant cataracts was 91.6% in BES, 96.3% in SINDI and 96.5% in SCES. Furthermore, our algorithm had a sensitivity of 88.8% with a specificity of 81.1% in BES, a sensitivity of 94.2% with a specificity of 90.3% in SINDI and a sensitivity of 96.0% with a specificity of 88.1% in SCES, for detection of visually significant cataracts (Table 2 and Fig. 1). At a moderate specificity level of 80%, the sensitivity for detecting visually significant cataracts was 98.8% in the internal test set and ranged from 85.7% to 98.9% in the external test sets (Table 3). The confusion matrices for all test sets (internal and external) are shown in Supplementary Fig. 1.

**Table 2:** Performance of classification algorithm in detection of visually significant cataracts

	Detection of visually significant cataracts^a^		
Testing sets	AUROC (%) (95% CI)	Sensitivity (%) (95% CI)	Specificity (%) (95% CI)
**Internal:**			
SIMES (*n* = 72; *N* = 1,692)	96.6 (95.5–97.7)	95.7 (90.5–100.0)	89.0 (84.7–93.5)
**External:**			
SCES (*n* = 141; *N* = 5,747)	96.5 (96.0–97.0)	96.0 (93.1–98.9)	88.1 (86.5–89.6)
SINDI (*n* = 138; *N* = 5,626)	96.3 (95.6–96.9)	94.2 (91.1–97.6)	90.3 (89.7–91.0)
BES (*n* = 48; *N* = 4,632)	91.6 (90.2–93.1)	88.8 (79.5–97.7)	81.1 (70.5–88.2)

^a^Cataract with BCVA < 20/60.

*n*, number of eyes with visually significant cataracts with BCVA cut-off of <20/60; *N*, total number of eyes.

**Table 3:** Sensitivity of classification algorithm in detection of visually significant cataracts^a^ at different specificity levels

Testing sets	Sensitivity (%) (95% CI)		
	At 70% specificity	At 80% specificity	At 90% specificity
**Internal**			
SIMES (*n* = 72; *N* = 1,692)	98.8 (97.6, 100.0)	98.8 (97.6, 100.0)	92.7 (87.2, 97.8)
**External**			
SCES (*n* = 141; *N* = 5,747)	100.0 (100.0,100.0)	98.9 (97.7, 100.0)	91.0 (87.2, 94.7)
SINDI (*n* = 138; *N* = 5,626)	97.7 (96.4, 98.9)	97.0 (95.2,98.9)	94.0 (90.8, 96.7)
BES (*n* = 48; *N* = 4,632)	95.3 (91.8, 97.9)	85.7 (80.4, 90.9)	71.5 (65.1, 77.8)

^a^Cataract with BCVA < 20/60.

*n*, number of eyes with visually significant cataracts with BCVA cut-off <20/60; *N*, total number of eyes

In a post-hoc subgroup analysis that further assessed the performance of the classification algorithm in the eyes of individuals aged ≥60 years (Supplementary Table 1), the AUROC for detection of visually significant cataracts was 93.3% (95% CI 91.1–95.4, sensitivity 90.5%, specificity 85.7%) in the internal test set. Across the external test sets, the AUROC ranged between 88.7% and 91.7%. In another post-hoc subgroup analysis by gender, similar performances to the results of the main analysis were observed (Supplementary Table 2).

Comparatively, when visually significant cataracts were defined based on a lower best-corrected visual acuity (BCVA) cut-off of <20/40^13^, we observed a similar, albeit slightly lower, performance of the algorithm. In the internal test set, the AUROC was 95.6% (95% CI 94.6–96.5), with a sensitivity of 91.5% and a specificity of 87.6%. When tested across the three external test sets, the AUROC of the algorithm was the highest in SINDI (95.5%), followed by SCES (95.2%) and BES (90.9%), (Supplementary Table 3 and Supplementary Fig. 2). Similarly, at a moderate specificity level of 80%, the sensitivity of the algorithm in detecting visually significant cataracts was 95.6% in the internal test set and in the range 88.7–96.4% in the external test sets (Supplementary Table 4).

We further assessed the performance of the algorithm for the detection of severe visually significant cataracts. In the internal test set, the AUROC was 97.2% (95% CI 96.4–98.0) with a sensitivity of 96.8% (95% CI 92.9–100.0) and a specificity of 90.4% (95% CI 83.6–91.7). Across the external test sets, the AUROC ranged from 90.0% to 97.3% (Supplementary Table 5).

In further subgroup analyses, we evaluated the performance of the algorithm in detecting visually significant cataracts among eyes of individuals with diabetes but with no vision-threatening DR. The AUROC was 94.7% (95% CI 91.4–97.9) in the internal test set, with a sensitivity of 93.8% (95% CI 80.0–100) and a specificity of 85.3% (95% CI 78.0–95.8). Across the external test sets, the AUROC ranged from 95.3% to 97.3% (Supplementary Table 6).

In another sensitivity analysis, we added back pseudophakic eyes (originally excluded in the main analysis) to the test sets of SIMES, SCES and SINDI. In this additional evaluation, visually significant posterior capsular opacification (PCO) (defined as pseudophakic eyes with concurrent PCO and BCVA < 20/60) were categorized as ‘ground truth positive’. The AUROC was 96.5% (95% CI 95.1–98.0) in the internal test set, with a sensitivity of 95.9% (95% CI 88.4–99.1%) and a specificity of 87.1% (95% CI 85.5–88.6%). The AUROC was 96.4% in the SCES external test set and 94.5% in the SINDI external test sets (Supplementary Table 7).

**Fig. 2:**
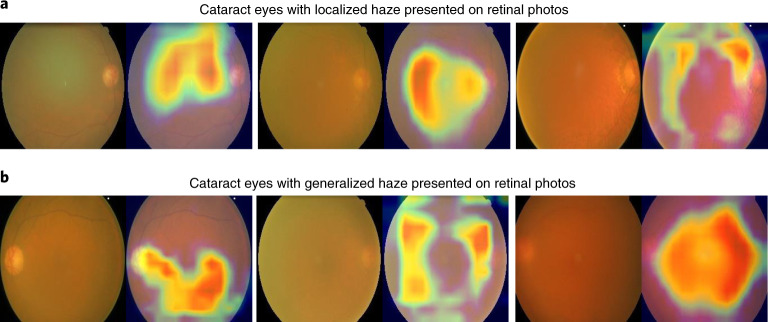
Saliency maps highlighting regions that the algorithm focuses on when predicting visually significant cataracts. The highlighted regions in retinal photographs are congruent with the pathological features that typically present in eyes with significant cataracts. Cataract eyes with localized haze (**a**) and generalized haze (**b**) presented on retinal photos.

In addition, we used saliency maps to provide insights into the regions in the fundus image that the algorithm most probably focused on when predicting the presence of visually significant cataracts. Based on the selective saliency maps shown in Fig. 2, we demonstrated that the regions probably used by the algorithm were congruent with haziness features on retinal images, typically associated with cataracts (as further confirmed by an ophthalmologist, T.H.R.).

We further investigated the causes of misclassifications committed by the algorithm in detecting visually significant cataracts. Among the 72 eyes with visually significant cataracts in the internal test dataset, the algorithm accurately identified 69 eyes (sensitivity of 95.7%). Nevertheless, the algorithm had missed three eyes and these false-negative classifications were associated with the early stages of a posterior subcapsular cataract (PSC; Supplementary Fig. 3).

On the other hand, in the internal test set, there was a total of 178 false-positive cases, of which 10 cases (5.6%) had BCVA > 20/60 and a relatively clear view of the fundus (Supplementary Fig. 4a), whereas the remaining 168 cases (94.4%) had moderate or significant ‘haziness’ on the retinal photo that was attributed to the presence of a cataract (Supplementary Fig. 4b). Saliency maps of false-positive examples from the internal test set are shown in Supplementary Fig. 5.

**Fig. 3:**
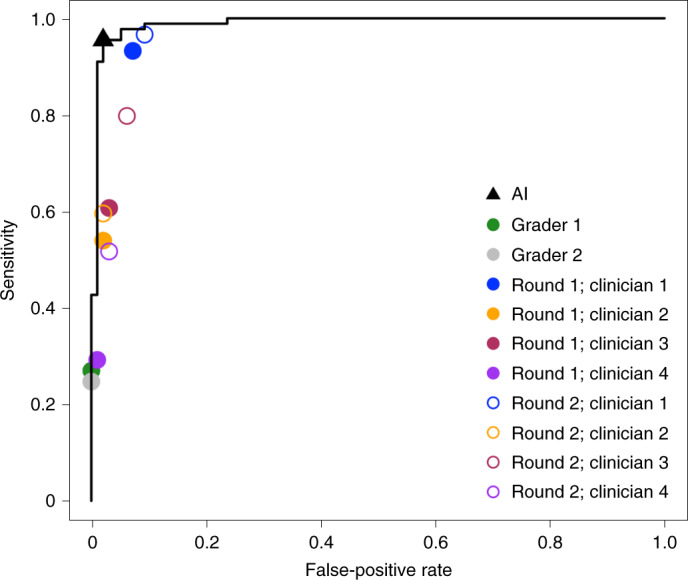
ROC curve showing performance of the algorithm versus 2 professional graders and 4 ophthalmologists on a test set of 186 eyes (randomly selected from SCES and SINDI).

To further evaluate our model performance, we tested our algorithm against two professional graders and four ophthalmologists in a subtest set of 186 randomly selected eyes. To illustrate our findings, Fig. 3 shows the performance of our algorithm versus professional ophthalmic graders and four ophthalmologists in a receiver operating characteristic (ROC) plot. In the first-round evaluation, in which only retinal images were used, the artificial intelligence algorithm achieved a sensitivity of 93.3% (95% CI 85.9–97.5%) and a specificity of 99.0% (95% CI 94.4–99.9%), outperforming most of the human experts (indicated as filled markers in Fig. 3). The two professional graders had sensitivity levels of 27.0% and 24.7%, and both had a specificity level of 100%. The four ophthalmologists had sensitivity levels ranging from 29.2% to 93.3% and specificity levels ranging from 92.8% to 99.0%. In the second-round evaluation, all four ophthalmologists re-evaluated the same set of retinal images but were further supplied with the corresponding slit-lamp photographs. Their performance improved (indicated as empty markers in Fig. 3), but most were still poorer than the algorithm. The sensitivity levels among the ophthalmologists’ second-round evaluation ranged from 51.7% to 96.6%, whereas the specificity levels ranged from 90.7% to 97.9%. A summary of the performance of the algorithm and human experts for the evaluations is shown in Table 4. Supplementary Fig. 6 further compares the number of inaccurate predictions (that is, error rate) between the algorithm and the human experts. In the first-round evaluation, the algorithm achieved an error rate of 3.8% (95% CI 1.5–7.6), significantly lower compared with the human experts (all comparisons *P* < 0.001, except for clinician 1 (*P* = 0.25)). The two professional graders had error rates of 34.9% (95% CI 28.1–42.3) and 36% (95% CI 29.1–43.4) respectively, whereas the four clinicians had error rates ranging from 7.0% (95% CI 3.8–11.7) to 34.4% (95% CI 27.6–41.7). In the second-round evaluation, the ophthalmologists’ error rates improved (ranging from 6.5% to 24.7%), but were still higher than the algorithm’s (all comparisons *P* ≤ 0.003, except for clinician 1 (*P* = 0.346)).

**Table 4:** Performance of AI and experts for the identification of visually significant cataract cases in a test set of 186 eyes

	Sensitivity (%) (95% CI)	Specificity (%) (95% CI)	TP (no.)	TN (no.)	FP (no.)	FN (no.)	Accuracy (%) (95% CI)	Error rate (%) (95% CI)
Round 1 (using retinal photographs only)								
Our algorithm	93.3 (85.9–97.5)	99.0 (94.4–99.9)	83	96	1	6	96.2 (92.4–98.5)	3.8 (1.5–7.6)
Grader 1	27.0 (18.1–37.4)	100.0 (96.3–100.0)	24	97	0	65	65.1 (57.7–71.9)	34.9 (28.1–42.3)
Grader 2	24.7 (16.2–35.0)	100.0 (96.3–100.0)	22	97	0	67	64.0 (56.6–70.9)	36.0 (29.1–43.4)
Clinician 1	93.3 (85.9–97.5)	92.8 (85.7–97.0)	83	90	7	6	93.0 (88.3–96.2)	7.0 (3.8–11.7)
Clinician 2	53.9 (43.0–64.6)	97.9 (92.7–99.7)	48	95	2	41	76.9 (70.2–82.7)	23.1 (17.3–29.8)
Clinician 3	60.7 (49.7–70.9)	96.9 (91.2–99.4)	54	94	3	35	79.6 (73.1–85.1)	20.4 (14.9–26.9)
Clinician 4	29.2 (20.1–39.8)	99.0 (94.4–100.0)	26	96	1	63	65.6 (58.3–72.4)	34.4 (27.6–41.7)
Round 2 (using both retinal and slit-lamp photographs)								
Clinician 1	96.6 (90.5–99.3)	90.7 (83.1–95.7)	86	88	9	3	93.5 (89.0–96.6)	6.5 (3.4–11.0)
Clinician 2	59.6 (48.6–69.8)	97.9 (92.7–99.7)	53	95	2	36	79.6 (73.1–85.1)	20.4 (14.9–26.9)
Clinician 3	79.8 (69.9–87.6)	93.8 (87.0–97.7)	71	91	6	18	87.1 (81.4–91.6)	12.9 (8.4–18.6)
Clinician 4	51.7 (40.8–62.4)	96.9 (91.2–99.4)	46	94	3	43	75.3 (68.4–81.3)	24.7 (18.7–31.6)

186 eyes randomly extracted from SCES and SINDI test sets, with visually significant cataracts defined as cataracts with BVCA < 20/60. Cataracts were graded based on the Wisconsin cataract grading system by A.G.T. independently, to form the gold standard for this evaluation.

TP, true positive; TN, true negative; FP, false positive; FN, false negative.

## Discussion

We developed and tested a deep-learning-based algorithm for the detection of visually significant, age-related cataracts, based on retinal photographs alone. When further compared with ophthalmologists’ evaluations, we demonstrated that the algorithm had a comparable, if not more superior, performance. Our findings indicate that this retinal photograph-based algorithm may be used as a simple, automated and potentially low-cost alternative for screening of visually significant cataracts among older adults. Against the backdrop of the growing number of cataracts globally due to aging populations, and a corresponding shortage of ophthalmologists^14^, this algorithm may help to improve the screening, identification and referral of appropriate patients for cataract surgery, especially in low-resourced communities.

The uniqueness of this work lies with the use of a single imaging modality (that is, only a macula-centered retinal photograph) for the detection of visually significant cataracts, unlike the ‘traditional’ method which requires both slit-lamp and retroillumination photographs alongside BCVA measurement. In the present study, we used large training and testing datasets consisting of 25,742 retinal photographs in total, curated from well-established, population-based studies (SIMES, SCES, SINDI and BES). Furthermore, we conducted external testing across three datasets (SCES, SINDI and BES), with the algorithm achieving an AUROC of >90% across all external datasets, demonstrating optimal generalizability of the algorithm. Across the external sets, the BES had a slightly lower area under the curve. This might be in part due to the different cataract grading system (Age-Related Eye Disease Study (AREDS)) and grader (J.B.J.) deployed in BES, compared with SCES and SINDI, which were both based on the Wisconsin grading system. In addition, BES had a smaller sample size and fewer visually significant cataract cases (*n* = 48).

In the subgroup analysis that assessed the performance of the algorithm among eyes of individuals aged ≥60 years, we observed largely similar performances across the internal and external test sets (Supplementary Table 2). This finding indicates that the algorithm would perform relatively well on older age groups with increased risk of cataracts^15^. In addition, among 49 cases of concurrent visually significant cataracts and retinal diseases (curated from the SIMES, SINDI and SCES test sets), our algorithm was able to correctly identify 46 of them (93.9%, results not shown in tables), indicating that the algorithm could potentially perform well even in the concurrent presence of other retinal pathologies. We also performed another sensitivity analysis with pseudophakic eyes (originally excluded in the main analysis) added back into the test sets of SIMES, SCES and SINDI. In this additional evaluation, visually significant PCO (defined as pseudophakic eyes with concurrent PCO and BCVA < 20/60) were categorized as ground truth positive as well. Overall, we observed that the algorithm’s performance in this evaluation was still largely similar to the results of the main analysis (Supplementary Table 7). However, it should be noted that only small numbers of visually significant PCO (12 in total) were available in the current test sets. Hence, the algorithm’s performance in the presence of pseudophakic and PCO eyes still requires future evaluation with larger samples of visually significant PCO.

As an extension of our primary evaluation, we further compared our algorithm’s performance with experts (professional ophthalmic graders and ophthalmologists). Based on retinal photographs alone, the algorithm achieved better performance (sensitivity of 93.3% and specificity of 99.0%) than all the experts. In a further evaluation in which the ophthalmologists were additionally provided with standard slit-lamp photographs to assess the human crystalline lens, the algorithm (with retinal photograph input alone) still outperformed most of the ophthalmologists, further highlighting the potential of the algorithm as a simple and automated detection tool for identifying visually significant cataract cases that probably warrant referrals for cataract surgery. Based on the algorithm’s sensitivity level of 93.3% in this test set of 186 eyes (89 positive cases), and the smallest difference of 3.3% between algorithm and human expert (clinician 1’s second-round performance), this sample of 186 eyes was sufficiently powered to confirm noninferiority (defined based on a 5% noninferiority margin) between algorithm and human expert, with a power of 95% at the 5% significance level.

Previous studies involving retinal images for cataract detection included relatively smaller datasets for the development of their algorithms, and most were not externally validated^8–12^. Importantly, the gold standard in these previous studies was based solely on a highly subjective method of classifying the retinal photographs’ haziness level (which could also be due to cornea opacity). Conversely, in our study, we used standardized and well-established cataract-grading protocols (the Wisconsin and AREDS grading systems)^16,17^. Furthermore, previous studies focused only on detecting the presence of cataracts, but without taking into account the visual function status (that is, whether there was substantial visual loss that further justified referral decision), which might inadvertently identify mild/nonvision-threatening cataract cases that typically do not require surgery in the short term, thus resulting in unnecessary/nonurgent referrals. In contrast, our developed algorithm was designed to identify visually significant cataract cases that would benefit more directly from cataract surgery.

Given that slit-lamp-based examinations and anterior segment photography (that is, of the exterior eye) are not commonly performed in community vision screening, and traditional measurement methods of BCVA (through subjective refraction or pinhole) require significant time, the conventional processes of determining visually significant cataracts in community screening involve multiple tests and skilled manpower. Moreover, conventional assessments based on anterior segment photographs, even when coupled with self-reported symptoms, would probably be insufficient for identifying the presence of visually significant cataracts. This is because cataracts are typically noticeable only from anterior evaluation when it is prominently dense or severe, and self-reported symptoms (such as glare or blur vision) are less accurate and may not necessarily be attributed to cataracts. Although some current screening programs screen and refer based on a best-corrected vision threshold only, it should be cautioned that this approach would not be able to definitively confirm the presence of cataracts, thus resulting in unnecessary false-positive referrals^18–20^. Importantly, such an approach would, at best, identify broad types of visual impairment cases that may not be cataract related, and thus would not fulfill the original purpose to specifically detect visually significant cataracts. Taken together, our proposed single-modality, retinal photograph-based algorithm could potentially offer a more efficient option for identifying visually significant cataract cases in the general population. Given the increasing accessibility of retinal photography and as it is already a routine procedure in most existing screening programs (for example, current DR-screening programs), the algorithm might be used as an add-on test with minimal additional cost. Compared with deployment among older adults, deployment in existing screening programs that are already equipped with a retinal camera might be more readily implementable in this context. In the same vein, in the additional subgroup analysis among people with diabetes but with no vision-threatening DR, we also observed similar algorithm performance to the main analysis (Supplementary Table 6), further supporting the notion of deploying the algorithm in existing DR-screening programs.

A secondary potential application would be on DR-screening programs in which cataracts are a common cause of ungradable retinal photographs^21–24^. An evaluation from the Thailand DR-screening program reported that ungradable retinal photographs affect DR-screening workflow, and participants with ungradable photos were instead referred directly to a secondary or tertiary eye hospital (Supplementary Fig. 7a, part i) which might inadvertently result in over-referrals^23^. In addition, based on the Singapore integrated DR-screening program’s (SIDRP’s) 2019 record, among 2,543 ungradable retinal photographs, 1,132 (44.5%) were due to media opacity, further highlighting the potential magnitude of cataract cases in DR-screening programs. In the SIDRP’s current workflow, in the event of an ungradable photograph, human grading would be deployed to further determine whether the nongradability is probably due to an artifact or significant cataract (Supplementary Fig. 7a, part ii). In this regard, our algorithm could possibly be deployed to ‘sieve out’ ungradable retinal photographs due to significant cataracts, thus making the current workflow less manpower intensive (as conceptually illustrated in Supplementary Fig. 7b). To demonstrate this potential utility, we randomly selected 305 cataract-suspected ungradable photographs from the SIDRP (where graders indicated cataract as the reason for referral, but in the absence of slit-lamp examination or photographs), and further tested our algorithm on this separate set. Of the 305, our algorithm identified 301 as being visually significant cataracts (98.7%, results not shown in tables), indicating that the algorithm may potentially improve current DR-screening program’s personnel-staffed workflow in identifying significant cataract cases among ungradable photos. Nevertheless, it should be noted that definite cataract diagnosis (that is, the required ground truth in this context) among these SIDRP patients could eventually be ascertained only by following through their referral path to the tertiary center. Such data are currently not available and require further data linkage with a tertiary hospital. These ground truth data would be important in serving as a gold-standard reference to compare the performance between the algorithm and the SIDRP’s human grader in the next real-world evaluation work.

Minimization of false-negative misclassifications is essential to avoid missing significant cataract cases that would benefit from cataract surgery. In this regard, we further evaluated the reasons for false-negative classifications in the internal test set (*n* = 3). The three false-negative cases had an early PSC, located centrally on the visual axis, thus affecting the vision significantly despite its small size (Supplementary Fig. 3). There were minimal haziness features on the retinal photograph, which might have led to the algorithm ‘missing’ such cases. When evaluating the false-negative cases in external test sets, similar observations were found (examples not shown in figures). Altogether, further refinement and training of the algorithm with the addition of these early, ‘on-axis’ PSC cases would possibly improve the algorithm’s performance further.

On the one hand, reduction of false-positive results is also important to avoid unnecessary referrals. In the internal test set, of the 178 false-positive eyes, 10 (5.6%) had a relatively clear fundus view with BCVA > 20/60, and were indeed falsely classified by our algorithm. On the other hand, 168 false-positive cases (94.4%) had a BCVA > 20/60 but actually presented with either a moderately or significantly hazy fundus (Supplementary Fig. 4). When evaluating false-positive cases in external test sets (based on 10% randomly selected from all false-positive cases in each external test set), similar observations were found (examples not shown in figures). Saliency maps among the false-positive cases consistently illustrated that the algorithm probably interpreted the haziness appearances on retinal photographs as the ‘features’ responsible for the ‘positive output’ prediction (Supplementary Fig. 5), indicating that these false-positive cases were not entirely random errors made by the algorithm. It should also be noted that, in some instances of dense cataract eyes, despite having relatively less affected BCVA, dense cortical or nuclear cataracts may still affect contrast sensitivity, resulting in compromised visual function or night vision^25,26^. Therefore, the above-mentioned false-positive cases of hazy-looking fundus, but without severely poor BCVA visual loss, would probably still benefit from referrals to tertiary centers, and may not be deemed entirely to be incorrect referrals. Nevertheless, this aspect still requires future testing and evaluations for further ascertainment.

Our study has several limitations. First, it should be noted that part of the ground truth definition of a visually significant cataract relied on BCVA measurement, which was dependent on the subject’s response during measurement, so measurement error in ground truth cannot be completely ruled out. Second, the slightly lower algorithm performance observed in the BES also highlighted the need to include more studies that utilized other cataract-grading protocols for future algorithm refinement. Last, despite the promising proof-of-concept demonstration, potential selection bias cannot be entirely ruled out because the examination setting, image types and qualities used in the present study may differ from the ones in the eventual deployment site. For future evaluation, it is important to further test the algorithm in a real-world community setting.

In conclusion, we developed and tested a retinal photograph-based, deep-learning algorithm for detection of visually significant age-related cataracts that allows automated and efficient referral to ophthalmologists for possible cataract surgery. This algorithm may potentially help to improve detection of visually significant cataracts in communities that lack trained eye-care personnel and resources.

## Methods

Participants’ written informed consent was obtained and the participants received reimbursement for their time in each study. All included studies adhered to the tenets of the Declaration of Helsinki and had respective local ethical committee approval. We obtained permission from the principal investigator of each study to use the data. This study followed the Strengthening the Reporting of Observational Studies in Epidemiology (STROBE) reporting guideline^27^.

### Study population

We developed and tested a deep-learning algorithm, using internal and external testing datasets comprising a total of 25,742 retinal photographs from 13,482 individuals across 4 studies. First, we utilized clinical data and retinal photographs of participants from the SIMES cohort as training sets^28–30^. The SIMES cohort dataset *(n* = 5,038; 9,737 eyes) was randomly distributed into a development set (*n* = 4,138) and an independent internal test set (*n* = 900; 1,692 eyes) based on an 8:2 ratio at the individual level (that is, by person). This was to ensure that there was no overlap of data from the same individual across the development and internal test sets, to prevent model overfitting. The internal test set was not accessed during model development.

We further used the following three datasets for external testing: the SINDI^31^, the SCES^31^ and the BES^32^. Among the included studies, the SIMES cohort, SINDI and SCES performed cataract grading from slit-lamp and retroillumination photographs based on the Wisconsin cataract grading system^16^, whereas the photographic cataract grading in the BES was based on the AREDS system^17^. Further details on these two cataract-grading protocols are described in Supplementary Fig. 8.

### Inclusion and exclusion criteria

Across the development and test sets, study participants with incomplete or missing cataract grading or BCVA data, or pseudophakic or aphakic eyes, were excluded. Study eyes with visual impairment caused by other pathologies such as DR, age-related macular degeneration and other maculopathy were also excluded. In the present study, only macula-centered retinal photographs were used. When multiple photographs were available for the same eye, only one photograph with the best quality was selected. Retinal photographs with severe motion or blinking artifacts and insufficient illumination were deemed to be poor quality due to artifact and were also excluded from the present study. Nevertheless, in photographs in which retinopathy cannot be graded due to media opacity (that is, ungradable photographs due to cataracts), they were still included for algorithm training. Further details on image exclusion are provided in Supplementary Table 8.

### Definition of visually significant cataracts

For the present study, eyes with cataracts were defined as eyes with any of the following: nuclear cataract at grade ≥4 according to the Wisconsin cataract grading system or grade ≥5 according to the AREDS system. Cortical cataracts were defined as ≥5% of total lens area involved with cortical opacity and PSC as any such opacity present (that is, >1%), in both grading systems. In SIMES, SCES and SINDI, the cataract was graded based on the Wisconsin grading system by a single grader with >15 years’ experience in performing Wisconsin cataract grading (A.G.T.). In the event of ambiguous cases, further adjudications were performed by a senior ophthalmologist (P.M.) and senior researcher (J.J.W.). In the BES, cataracts were graded based on the AREDS system and performed by a senior ophthalmologist (J.B.J.).

Visually significant cataracts were then defined as cataract eyes with BCVA < 20/60 (the World Health Organization’s definition for low vision)^13^. Eyes with severe visually significant cataracts were defined as late-stage cataracts (cortical cataract ≥25% or PSC > 5% or nuclear cataract ≥grade 4 (Wisconsin)) with concurrent BCVA< 20/60.

### Development of deep-learning system

In the present study, we adopted a supervised deep-learning approach to developing a classification-based, deep-learning model for the detection of visually significant cataracts. The primary inputs to the deep-learning model included preprocessed macula-centered retinal photographs and clinical labels (that is, visually significant cataract status). All retinal photographs were resized to dimensions of 224 × 224 pixels. Within the development set (80% randomly selected from the SIMES cohort), a fivefold crossvalidation was performed for fine-tuning of the model hyperparameters.

Overall, the framework of our algorithm comprises two parts: a deep convolutional neural network (CNN) serving as a feature extractor and a classification model (Supplementary Fig. 9). Specifically, a deep CNN, namely, the residual neural network (ResNet)-50, was first used to extract features from the retinal photographs^33^. The ResNet-50 had been pretrained on the ImageNet dataset^34^. The training retinal images were fed to the CNN model to extract their features, a process referred to as ‘feature extraction’. In this instance, 2,048 features were extracted from each training image. These extracted features, along with the ground truth clinical labels, were then used to classify the image through a classification model (XGBoost classifier) in which we applied an extreme gradient-boosting technique with the use of a scalable tree-boosting system^35^. The XGBoost method was based on a gradient-boosting approach, in which decision trees were gradually added, such that each subsequent tree reduced the error of the preceding ones^35^. This method aimed to prevent overfitting using the regularization techniques, parallelized tree building, tree pruning and other enhancement features^35^. The parameters for the XGBoost classifier, such as learning rate, minimum sum of instance weight needed, maximum depth of the tree and number of estimators, were chosen using the grid-search approach^35^, to minimize its crossvalidated classification error in the development set. In addition, as the dataset was imbalanced, we also adjusted the classifier parameters to balance the impact of positive and negative samples. Once the model had been trained, it was used for making predictions on the independent (that is, nonoverlapping with the development set) internal and external test datasets. The final output of the classification-based model was the probability for the presence of visually significant cataracts in each study eye. Details of the model development are described in Supplementary Note.

### Comparison in performance with clinical experts

From external datasets of the SCES and SINDI, we further extracted a subtest set of 186 eyes, which consisted of 97 randomly selected eyes with nonvisually significant cataracts (selected from all negative cases), and 89 eyes with visually significant cataracts (selected from all eyes with visually significant cataracts). For each eye, we obtained referral suggestions from six clinical experts, including two professional graders (not including A.G.T. who performed the ground truth cataract grading for the SCES and SINDI datasets, and established the gold-standard reference for this evaluation) and four ophthalmologists (years of experience ranged from 1 year to 7 years). In the initial round of evaluation, all experts were presented with just the retinal images. During the second round of evaluation, the four clinicians were presented with the same set of retinal images, reshuffled, but with additional slit-lamp photographs of each eye. The graders and our algorithm received only retinal images and were not involved in the second round of evaluation. We compared each of these performances (our algorithm and clinical experts) against the gold-standard diagnosis of a visually significant cataract, as defined in the previous section.

### Saliency map

For better understanding of which regions of the retinal photographs were more likely to be used by the algorithm for prediction of normal eyes or eyes with visually significant cataract, we used the GradCAM method to generate saliency maps^36^. With these saliency maps presented as colored heatmaps, regions with greater contributions to the predicted output were highlighted with a ‘hotter’ color on the heatmaps. The saliency maps were resized to 224 × 224 pixels^2^ and layered over the retinal images. Details of the saliency map generation method have been described in Supplementary Note.

### Statistical analysis

To evaluate the performance of the algorithm for binary classification of visually significant cataracts, we used AUROC, sensitivity and specificity. The classification threshold was selected based on Youden’s index^37^. We calculated the 95% CI for these performance measures, using 2,000 bootstrap replicates. We performed the statistical analyses using standard statistical software (STATA, v.16, Texas; R v.1.1.456).

To compare the performance of the algorithm with that of the group of clinical experts, we used metrics of sensitivity, specificity, accuracy and error rate. Accuracy was defined as the percentage of the total number of accurate predictions (that is, sum of true-positive and true-negative cases) out of the total number of predictions. The error rate was then calculated as 1 − accuracy.

### Reporting summary

Further information on research design is available in the Nature Research Reporting Summary linked to this article.

## Supplementary information

Supplementary Information Supplementary Figs. 1–9, Tables 1–8. Details on model development and saliency map generation

Reporting Summary

## Data Availability

We used TensorFlow (v.1.14.0) for development of the algorithms, including packages such as Torch (v.1.8.0), Torchvision (v.0.9.0), OpenCV (v.3.4.3.18), scikit-learn (v.0.20.02) and XGBoost (v.0.82). The testing code used in the present study can be accessed at 10.5281/zenodo.5650719. As the optimized algorithm is currently undergoing patent examination process, customized codes can be made available for research purpose from the corresponding author (C.-Y.C.) upon reasonable request. All requests for code will be reviewed by the SingHealth Intellectual Property Unit, to verify whether the request is subject to any intellectual property or confidentiality constraints. Any code that can be shared will be released via a Material Transfer Agreement for noncommercial research purposes under the Creative Commons Attribution NonCommercial-NoDerivatives 4.0 license.

## Data availability

The main data supporting the results in the present study are available within the paper and its supplementary information. The retinal images and patient information are not publicly available due to patient privacy and the data are meant for research purposes only. On reasonable request, de-identified individual-participant data from the SIMES, SCES and SINDI datasets may be made available for academic purposes from the corresponding author (C.-Y.C.), subject to permission from the local institutional review board. Any data that can be shared will be released via a Material Transfer Agreement for noncommercial research purposes. Data from the BES dataset cannot be readily released due to patient privacy and the data are meant for research use only. Reasonable requests for data from the BES cohort should be made directly to J.J. (email: jost.jonas@medma.uni-heidelberg.de) for consideration. Data can be made available for research purposes, subject to permission from the local institutional review board.
