# Author Correction: Detecting visually significant cataract using retinal photograph-based deep learning

**DOI:** 10.1038/s43587-022-00245-5

**Published:** 2022-06-10

**Authors:** Yih-Chung Tham, Jocelyn Hui Lin Goh, Ayesha Anees, Xiaofeng Lei, Tyler Hyungtaek Rim, Miao-Li Chee, Ya Xing Wang, Jost B. Jonas, Sahil Thakur, Zhen Ling Teo, Ning Cheung, Haslina Hamzah, Gavin S. W. Tan, Rahat Husain, Charumathi Sabanayagam, Jie Jin Wang, Qingyu Chen, Zhiyong Lu, Tiarnan D. Keenan, Emily Y. Chew, Ava Grace Tan, Paul Mitchell, Rick S. M. Goh, Xinxing Xu, Yong Liu, Tien Yin Wong, Ching-Yu Cheng

**Affiliations:** 1 Singapore Eye Research Institute, Singapore National Eye Centre, Singapore, Singapore; 2 Duke-NUS Medical School, Singapore, Singapore; 3 Department of Ophthalmology, Yong Loo Lin School of Medicine, National University of Singapore, Singapore, Singapore; 4 Institute of High Performance Computing, A*STAR, Singapore, Singapore; 5 Beijing Institute of Ophthalmology, Beijing Ophthalmology and Visual Science Key Lab, Beijing, China; 6 Department of Ophthalmology, Medical Faculty Mannheim of the Ruprecht-Karis-University Heidelberg, Mannheim, Germany; 7 National Center for Biotechnology Information, National Library of Medicine, National Institutes of Health, Bethesda, MD USA; 8 National Eye Institute, National Institutes of Health, Bethesda, MD USA; 9 Centre for Vision Research, Department of Ophthalmology, The Westmead Institute for Medical Research, University of Sydney, Westmead Hospital, Westmead, New South Wales Australia; 10 National Health Medical Research Council Clinical Trials Centre, University of Sydney, Sydney, New South Wales Australia; 11 Department of Ophthalmology, Yong Loo Lin School of Medicine, National University of Singapore, Singapore, Singapore

**Keywords:** Diagnostic markers, Predictive markers, Ageing

Correction to: *Nature Aging* 10.1038/s43587-022-00171-6, published online 21 February 2022

This paper was originally published under standard Springer Nature license (© The Author(s), under exclusive licence to Springer Nature America, Inc.). It is now available as an Open Access paper under a Creative Commons Attribution 4.0 International license, © The Author(s). In addition, a new affiliation (Department of Ophthalmology, Yong Loo Lin School of Medicine, National University of Singapore, Singapore, Singapore) has been added for Yih-Chung Tham, and the Acknowledgements have been amended to include the text “This project is supported by the Agency for Science, Technology and Research (A*STAR) under its RIE2020 Health and Biomedical Sciences (HBMS) Industry Alignment Fund Pre-Positioning (IAF-PP) grant no. H20c6a0031. Any opinions, findings and conclusions or recommendations expressed in this material are those of the author(s) and do not reflect the views of the A*STAR.” The changes have been made to the HTML and PDF versions of the article.

