# Supplementary material for: Detecting visually significant cataract using retinal photograph-based deep learning
Source: Nat Aging. 2022 Feb 21;2(3):264–71. doi: 10.1038/s43587-022-00171-6 (PMC10154193; doi:10.1038/s43587-022-00171-6)
Supplement: Supplementary file 1 — Supplementary Information Supplementary Figs. 1–9, Tables 1–8. Details on model development and saliency map generation [file 43587_2022_171_MOESM1_ESM.pdf]

nature portfolio

https://doi.org/10.1038/s43587-022-00171-6

Supplementary information

Detecting visually significant cataract using retinal photograph-based deep learning

In the format provided by the authors and unedited

Supplementary Tables

Supplementary Table 1: Performance of Classification Model in Detection of Visually Significant Cataract (defined based on BCVA of worse than 20/60) among eyes of individuals aged 60 years and above.

	**Detection of visually significant cataract**		
**Test sets**	**AUROC % (95% CI)**	**Sensitivity % (95% CI)**	**Specificity % (95% CI)**
**Internal:**			
**SIMES**	93.3 (91.1 - 95.4)	90.5 (83.7 - 97.4)	85.7 (82.0 - 87.7)
**External:**			
**SCES**	91.7 (90.6 - 92.9)	91.9 (85.3 – 98.0)	77.7 (72.3 - 82.5)
**SINDI**	91.6 (90.2 - 93.1)	89.2 (83.3 - 93.1)	84.1 (82.1 -89.2)
**BES**	88.7 (86.9 - 90.5)	86.0 (80.0 - 95.1)	80.3 (65.9 - 81.6)

SIMES, Singapore Malay Eye Study; SCES, Singapore Chinese Eye Study; SINDI, Singapore Indian Eye Study; BES, Beijing Eye Study.

Supplementary Table 2: Performance of Classification Model in Detection of Visually Significant Cataract (defined based on BCVA of worse than 20/60) by gender.

Test sets	Among eyes of male individuals			Among eyes of female individuals		
	**AUROC % (95% CI)**	**Sensitivity % (95% CI)**	**Specificity % (95% CI)**	**AUROC % (95% CI)**	**Sensitivity % (95% CI)**	**Specificity % (95% CI)**
**Internal:**						
**SIMES**	97.5 (96.5 - 98.5)	100.0 (95.0 -100.0)	91.4 (84.8 - 94.9)	95.5 (93.6 - 97.5)	95.5 (86.4 - 100.0)	89.1 (81.9 - 93.3)
**External:**						
**SCES**	96.3 (95.5 – 97.0)	97.1 (91.7- 100.0)	87.2 (83.5 - 90.5)	96.7 (96.0 - 97.4)	97.4 (92.3 - 100.0)	89.4 (83.4 - 90.5)
**SINDI**	94.5 (93.1 - 95.7)	91.4 (85.7 - 96.9)	90.3 (89.6 - 91.2)	97.3 (96.5 - 98.1)	95.1 (91.4 - 100.0)	91.3 (90.7 - 95.0)
**BES**	87.5 (84.6 - 90.5)	88.9 (70.6 - 95.0)	76.9 (68.8 - 92.6)	94.1 (92.7 - 95.5)	92.9 (88.5 - 96.6)	88.3 (87.6 - 88.9)

SIMES, Singapore Malay Eye Study; SCES, Singapore Chinese Eye Study; SINDI, Singapore Indian Eye Study; BES, Beijing Eye Study.

Supplementary Table 3: Performances of Classification Algorithm in Detection of Visually Significant Cataract (with BCVA cut-off of <20/40).

	Detection of visually significant cataract		
**Test sets**	**AUROC % (95% CI)**	**Sensitivity % (95% CI)**	**Specificity % (95% CI)**
**Internal:**			
SIMES (n=133, N=1,692)	95.6 (94.6, 96.5)	91.5 (86.7, 96.2)	87.6 (82.9, 92.6)
**External:**			
SCES (n=384, N=5,747)	95.2 (94.5, 95.8)	93.1 (89.4, 96.2)	85.0 (81.6, 88.6)
SINDI (n=268, N=5,626)	95.5 (94.9, 96.1)	90.4 (86.2, 97.1)	88.5 (81.2, 92.2)
BES (n=231, N= 4,632)	90.9 (89.6, 92.3)	89.2 (81.9, 93.4)	80. 5 (77.0, 87.1)

*Cataract with best-corrected visual acuity (BCVA) of worse than 20/40.

n, number of eyes with visually significant cataract with BCVA cut-off of <20/40; N, total number of eyes

SIMES, Singapore Malay Eye Study; SCES, Singapore Chinese Eye Study; SINDI, Singapore Indian Eye Study; BES, Beijing Eye Study.

Supplementary Table 4: Sensitivity of Classification Algorithm in Detection of Visually Significant Cataract (with BCVA cut-off of <20/40), at Different Specificity Levels.

Testing sets	Sensitivity (%)		
	**At 70% specificity**	**At 80% specificity**	**At 90% specificity**
**Internal:**			
SIMES (n=133, N=1,692)	99.4 (98.6 – 100.0)	95.6 (92.5 – 98.8)	85.9 (81.6 – 90.3)
**External:**			
SCES (n=384, N=5,747)	98.7 (97.8 – 99.6)	96.0 (94.4 – 97.5)	85.0 (82.0 – 88.1)
SINDI (n=268, N=5,626)	98.3 (97.1 – 99.4)	96.4 (94.6 – 98.1)	87.9 (85.3 – 90.6)
BES (n=231, N=4,632)	92.0 (89.3 – 94.8)	88.7 (85.3 – 92.1)	74.6 (71.0 – 78.3)

n, number of eyes with visually significant cataract with BCVA cut-off of <20/40; N, total number of eyes

SIMES, Singapore Malay Eye Study; SCES, Singapore Chinese Eye Study; SINDI, Singapore Indian Eye Study; BES, Beijing Eye Study.

Supplementary Table 5: Performances of Classification Algorithm in Detection of Severe Visually Significant Cataract.

**Test sets**	**Detection of severe visually significant cataract***		
	**AUROC % (95% CI)**	**Sensitivity % (95% CI)**	**Specificity % (95% CI)**
**Internal:**			
SIMES (n= 61, N=1,692)	97.2 (96.4, 98.0)	96.8 (92.9, 100.0)	90.4 (83.6, 91.7)
**External:**			
SCES (n= 116, N=5,747)	97.3 (96.8, 97.8)	95.6 (91.9, 100.0)	90.2 (87.1, 92.4)
SINDI (n= 118, N=5,626)	96.4 (95.9, 97.0)	94.2 (90.9, 97.3)	91.9 (90.3, 92.8)
BES (n= 43, N=4,630)	90.0 (88.0, 92.0)	84.9 (77.8, 94.0)	85.3 (76.5, 89.3)

*Defined as eyes with concurrent late stage cataract (i.e. cortical cataract ≥ 25% or PSC ≥ 5% or nuclear cataract ≥ grade 4 [Wisconsin cataract grading system]) and best-corrected visual acuity of worse than 20/60.

n, number of eyes with severe visually significant cataract; N, total number of eyes.

SIMES, Singapore Malay Eye Study; SCES, Singapore Chinese Eye Study; SINDI, Singapore Indian Eye Study.

Supplementary Table 6: Performance of Classification Model in Detection of Visually Significant Cataract (defined based on BCVA of worse than 20/60) among eyes of diabetic individuals without diabetic retinopathy.

	**Among diabetic individuals without diabetic retinopathy**		
**Test sets**	**AUROC % (95% CI)**	**Sensitivity % (95% CI)**	**Specificity % (95% CI)**
**Internal:**			
SIMES (n=27, N=456)	94.7 (91.4 - 97.9)	93.8 (80.0 - 100.0)	85.3 (78.0 - 95.8)
**External:**			
SCES (n=41, N=871)	97.3 (96.4 - 98.1)	96.4 (89.7- 100.0)	91.7 (83.3 - 95.5)
SINDI (n=83, N=1,948)	96.2 (95.3 - 97.1)	95.8 (89.8 - 100.0)	87.6 (85.9 - 93.1)
BES (n=9, N=557)	95.3 (92.8 - 96.7)	100.0 (100.0 - 100.0)	88.5 (87.1 - 89.7)

n, number of eyes with visually significant cataract; N, total number of eyes.

SIMES, Singapore Malay Eye Study; SCES, Singapore Chinese Eye Study; SINDI, Singapore Indian Eye Study; BES, Beijing Eye Study.

Supplementary Table 7: Performance of algorithm for the detection of visually significant cataract and posterior capsular opacification* (pseudophakic eyes included in this evaluation)

Testing sets	AUROC %	Sensitivity %	Specificity %
**Internal:**			
SIMES (n= 73, N= 1,843)	96.5 (95.1 - 98.0)	95.9 (88.4 - 99.1)	87.1 (85.5 - 88.6)
**External:**			
SCES (n= 142, N= 6,392)	96.4 (95.3 - 97.4)	95.1 (90.1 - 98.0)	88.0 (87.2 - 88.8)
SINDI (n= 148, N= 6,356)	94.5 (92.7 - 96.3)	87.2 (80.7 - 92.1)	90.7 (89.9 - 91.4)

*Defined as eyes with cataract or posterior capsule opacification (PCO) with best-corrected visual acuity of worse than 20/60.

n, number of eyes with visually significant cataract or PCO; N, total number of eyes.

SIMES, Singapore Malay Eye Study; SCES, Singapore Chinese Eye Study; SINDI, Singapore Indian Eye Study.

Supplementary Table 8: Details on Image Exclusion from Each Dataset.

	Number of images before exclusion	Number of images after exclusion	Number of images excluded (%)
**Development & internal test set:**			
SIMES	10,362	9,737	624 (6.0%)
**External test set:**			
SCES	6,568	5,747	821(12.5%)
SINDI	6,500	5,626	874 (13.4%)
BES	5,197	4,632	565 (10.9%)

SIMES, Singapore Malay Eye Study; SCES, Singapore Chinese Eye Study; SINDI, Singapore Indian Eye Study; BES, Beijing Eye Study.

Supplementary Tables

Supplementary Figure 1 Confusion matrices for the detection of visually significant cataract in all test sets (based on BCVA cut-off of worse than 20/60).

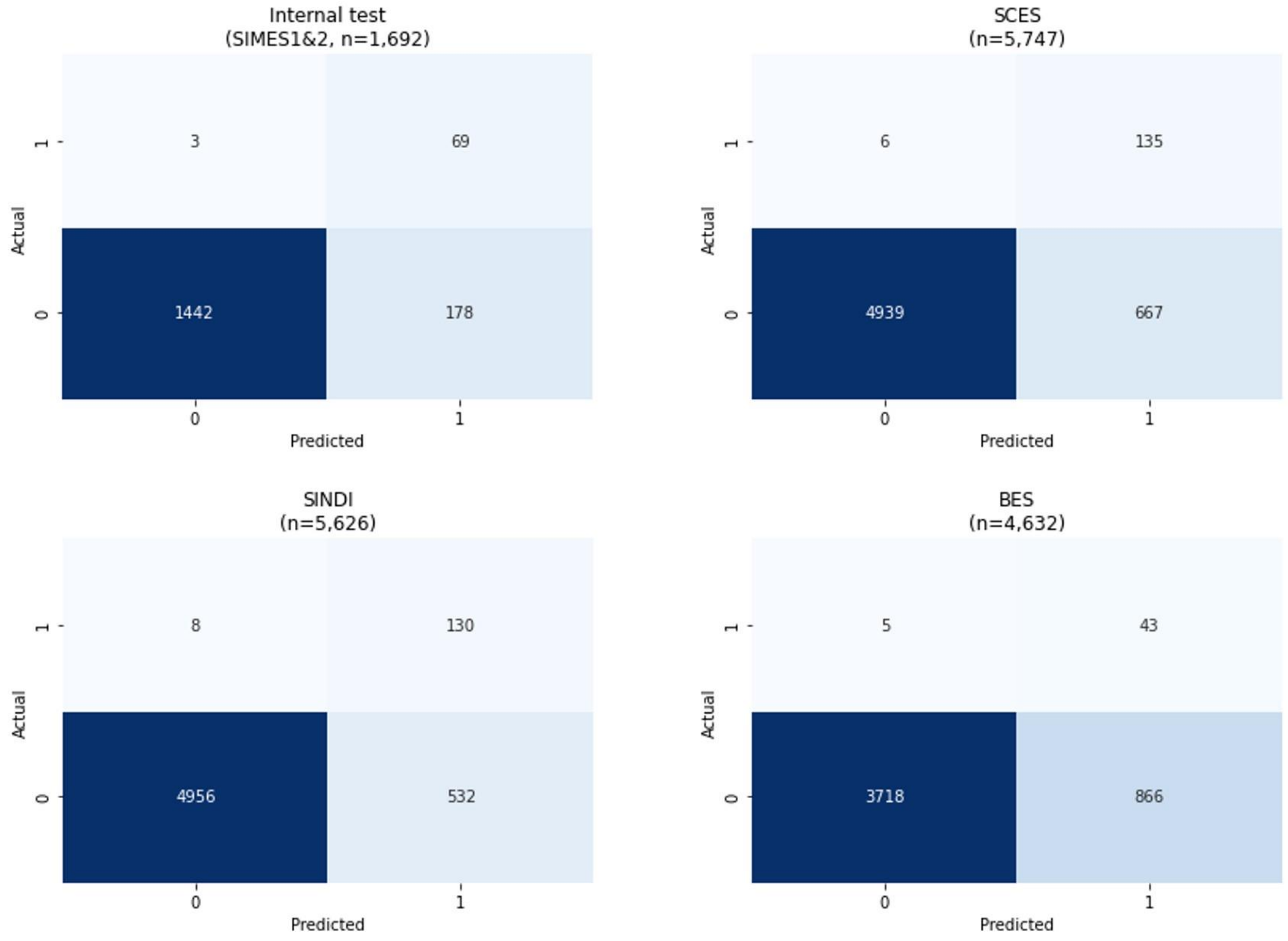

Supplementary Figure 2: ROC curve showing performance of the classification algorithm for the detection of visually significant cataract (based on BCVA cut-off of worse than 20/40).

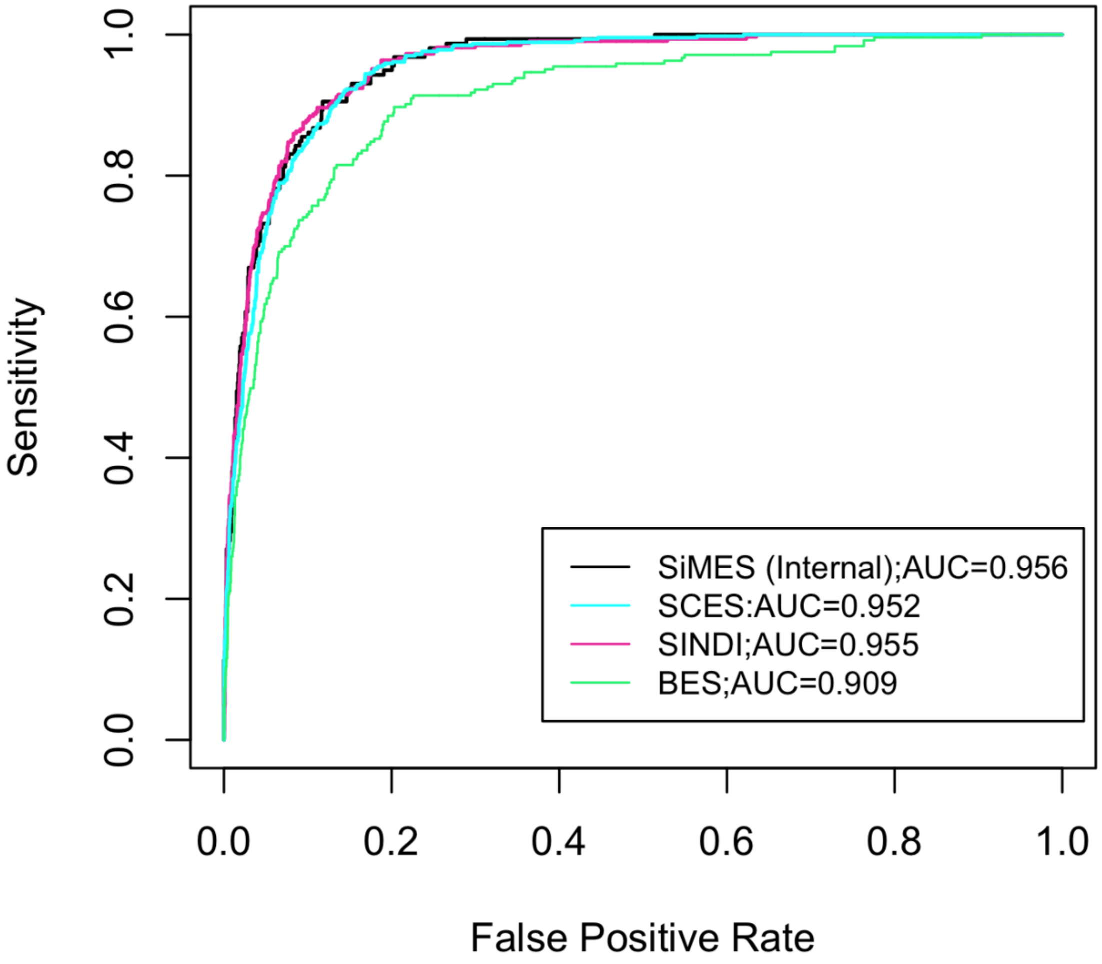

Supplementary Figure 3: Example of false negative classifications identified by the algorithm.

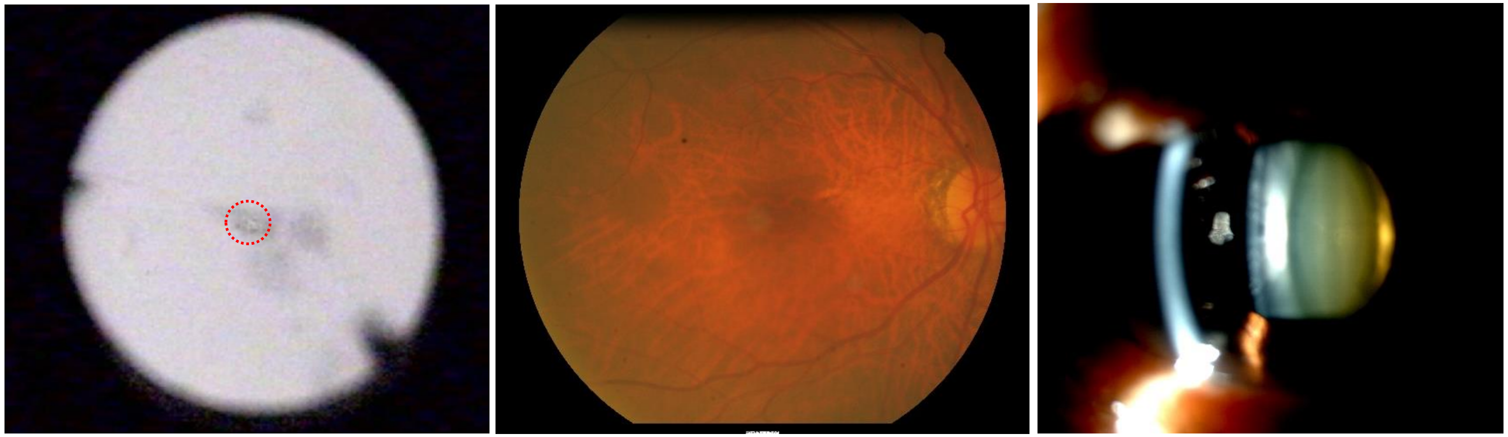

Nuclear grade 4, early cortical cataract (6.1%) and PSC (1.2%), BCVA = 20/80^{+1}

*Red dotted circle indicates PSC located centrally along visual axis.

Supplementary Figure 4: Examples of false positive classifications identified by the algorithm.

A) Relatively clear fundus view with BCVA better than 20/60

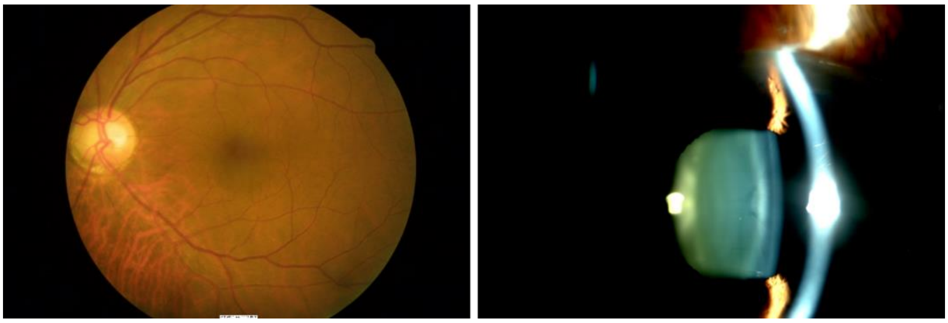

Nuclear grade 3, cortical cataract (3.8%), no PSC (0%), BCVA = 20/25

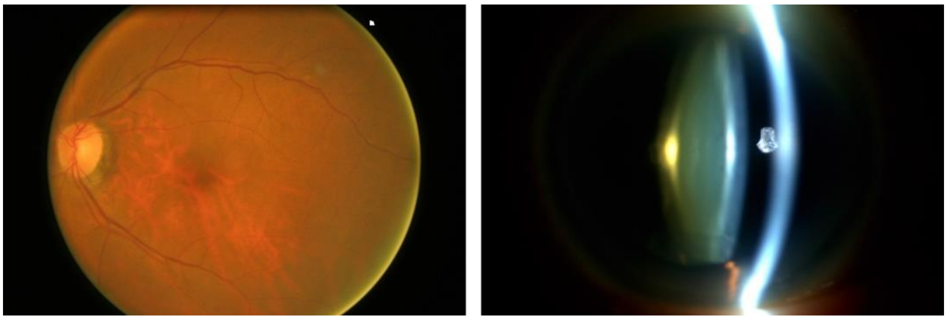

Nuclear grade 3, early cortical cataract (7.8%), no PSC (0%), BCVA = $20/16^{-2}$

B) Hazy fundus view, but BCVA better than 20/60

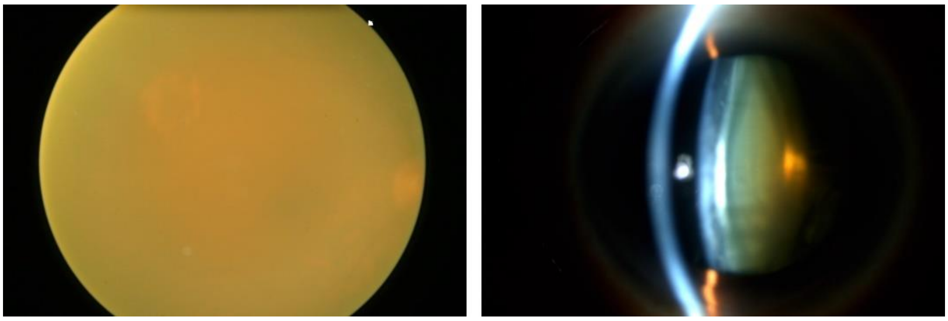

Nuclear grade 4, late cortical cataract (30.2%), no PSC (0%), BCVA= $20/40^{-1}$

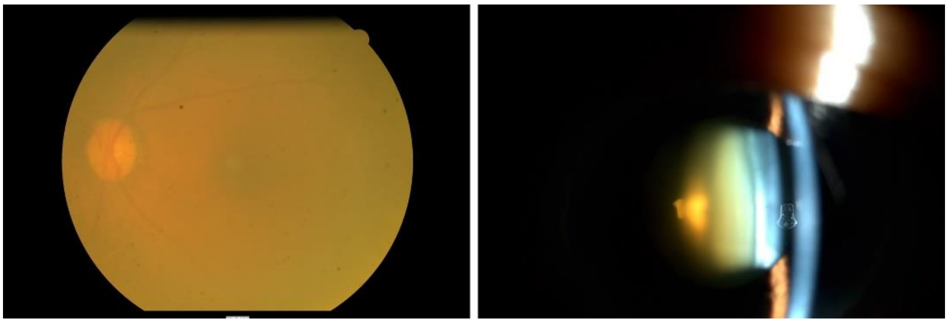

Nuclear grade 4, no cortical cataract (0%), late PSC (12.1%), BCVA=$20/63^{+2}$

Supplementary Figure 5: Saliency maps of false positive classifications made by the algorithm.

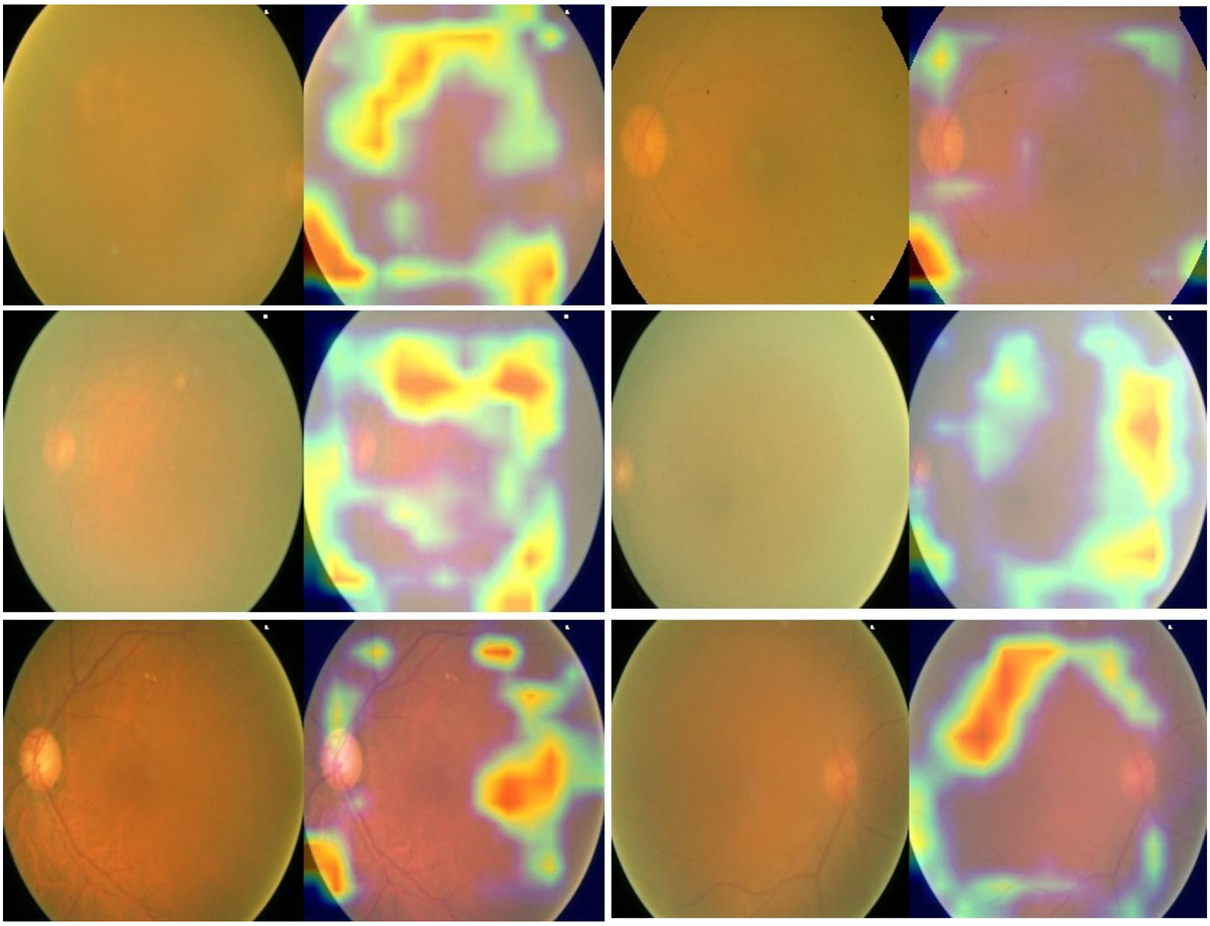

Supplementary Figure 6 Comparison of performances on classification of visually significant cataract between the algorithm and 6 clinical experts, assessed based on error rate % (1 – accuracy rate). Six clinical experts include two professional graders and four ophthalmologists (years of experience ranged from 1 to 7 years).

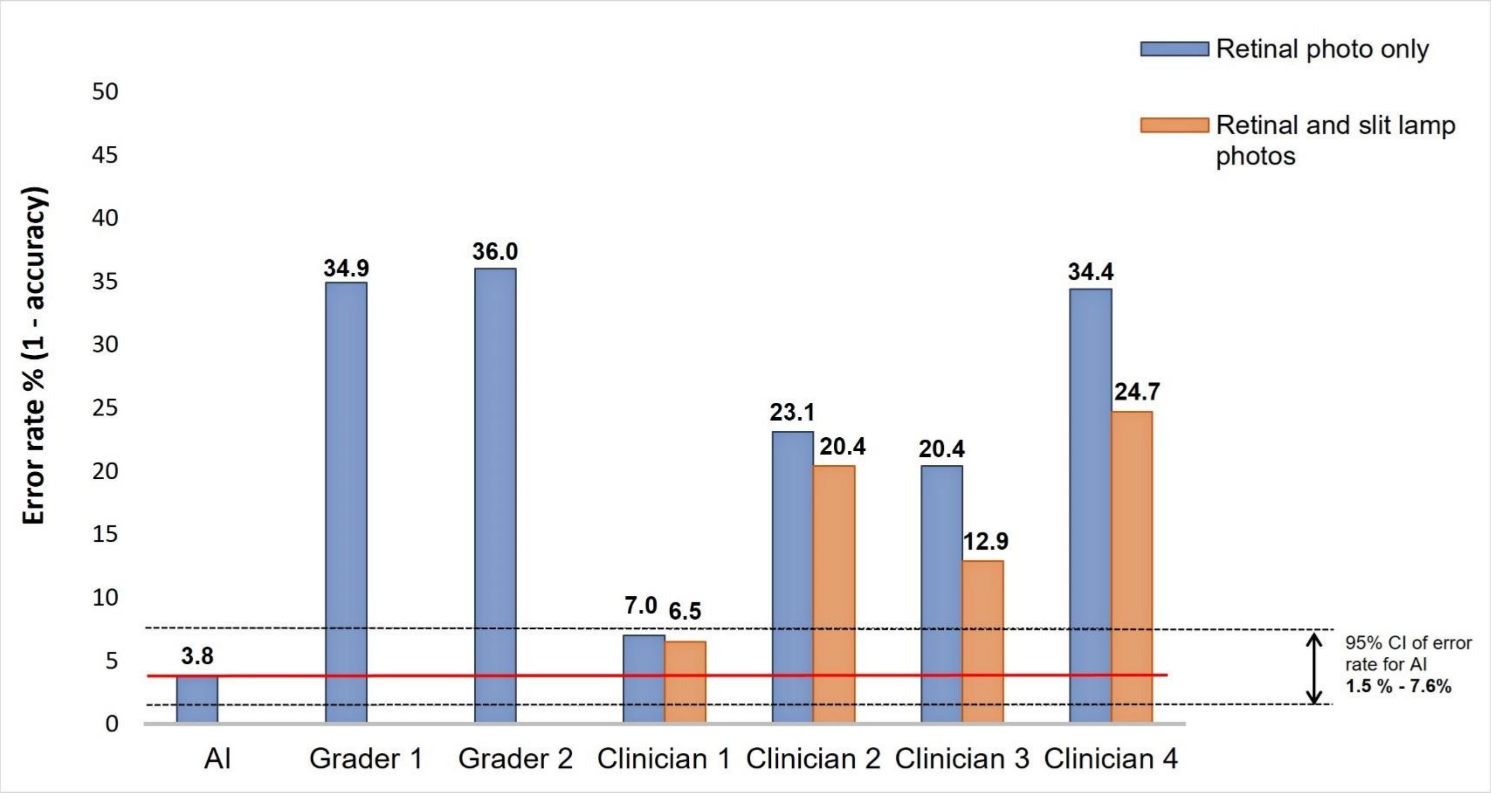

Supplementary Figure 7: Future possible deployment scenario of the algorithm in existing DR screening workflow.

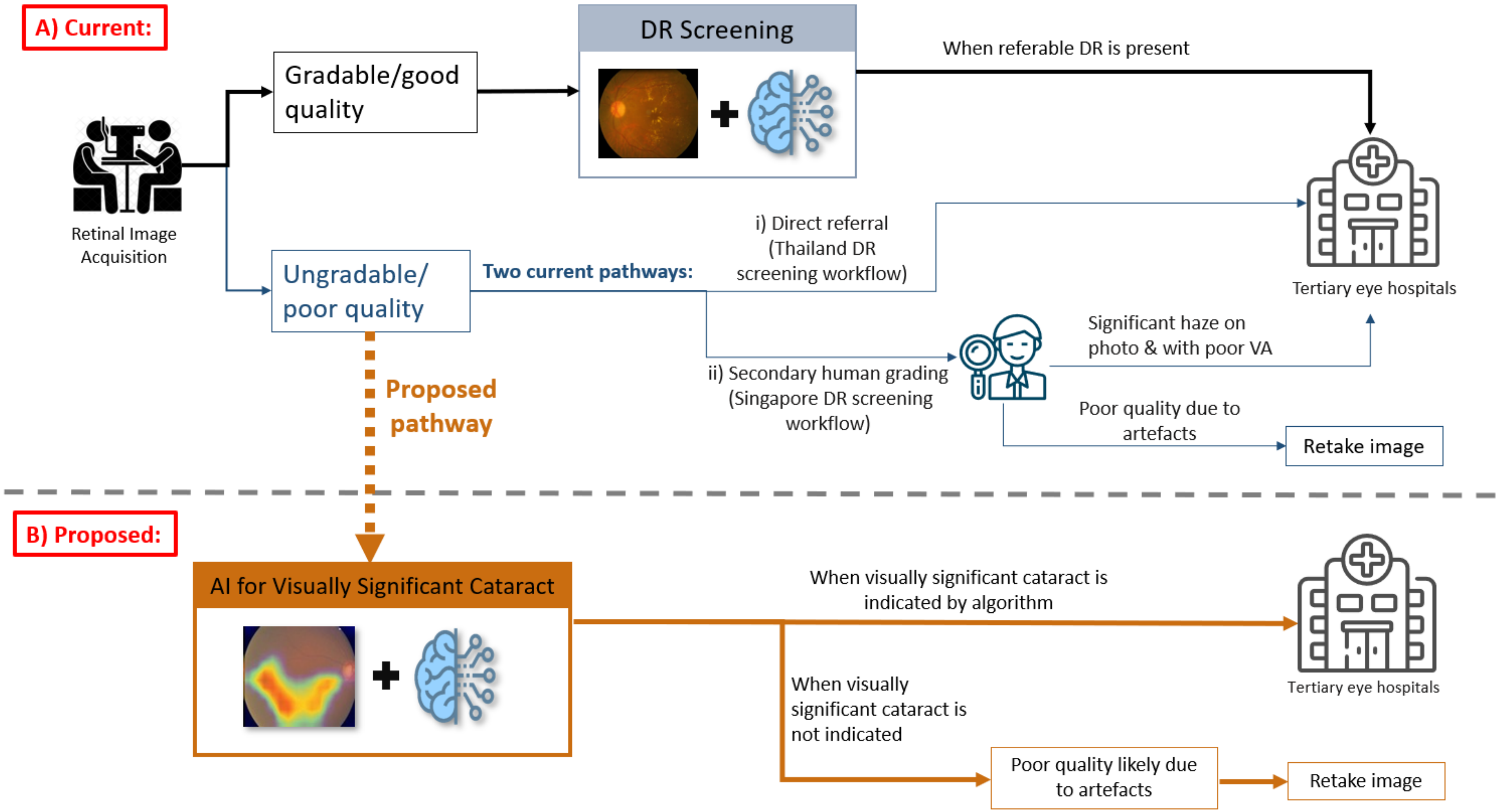

Supplementary Figure 8: Comparison between the AREDS and Wisconsin cataract grading systems. **A)** For nuclear cataract grading, different standard photographs were used in both systems; **B)** For cortical and posterior subcapsular cataract, same grading protocol was used in both systems, which involved anterior retro-illumination photograph with a measurement grid (with three concentric circles) placed on top of the photograph. *Horizontal red line denotes the cut-off that was used to define significant nuclear cataract in both systems.

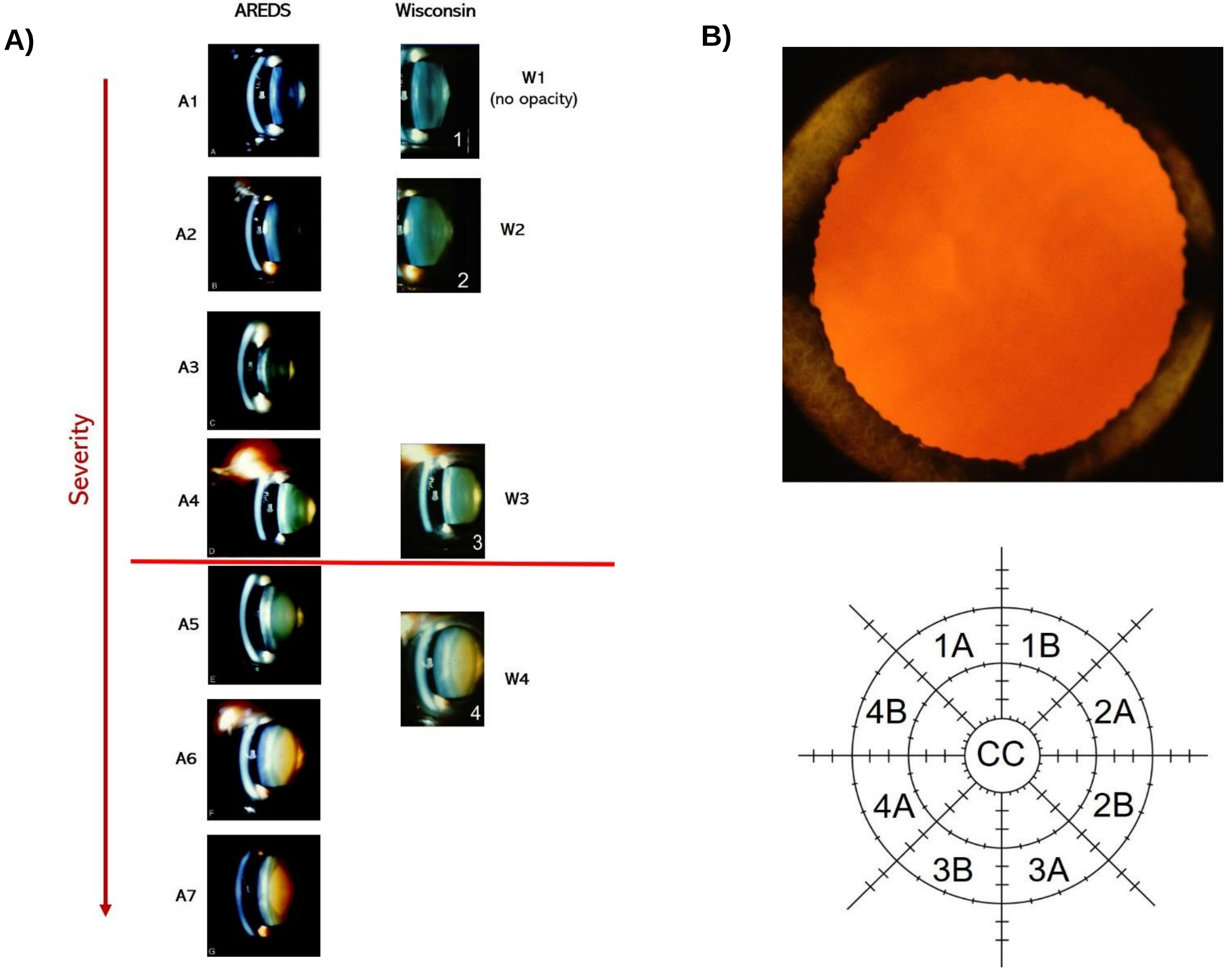

Supplementary Figure 9: Overview of model framework

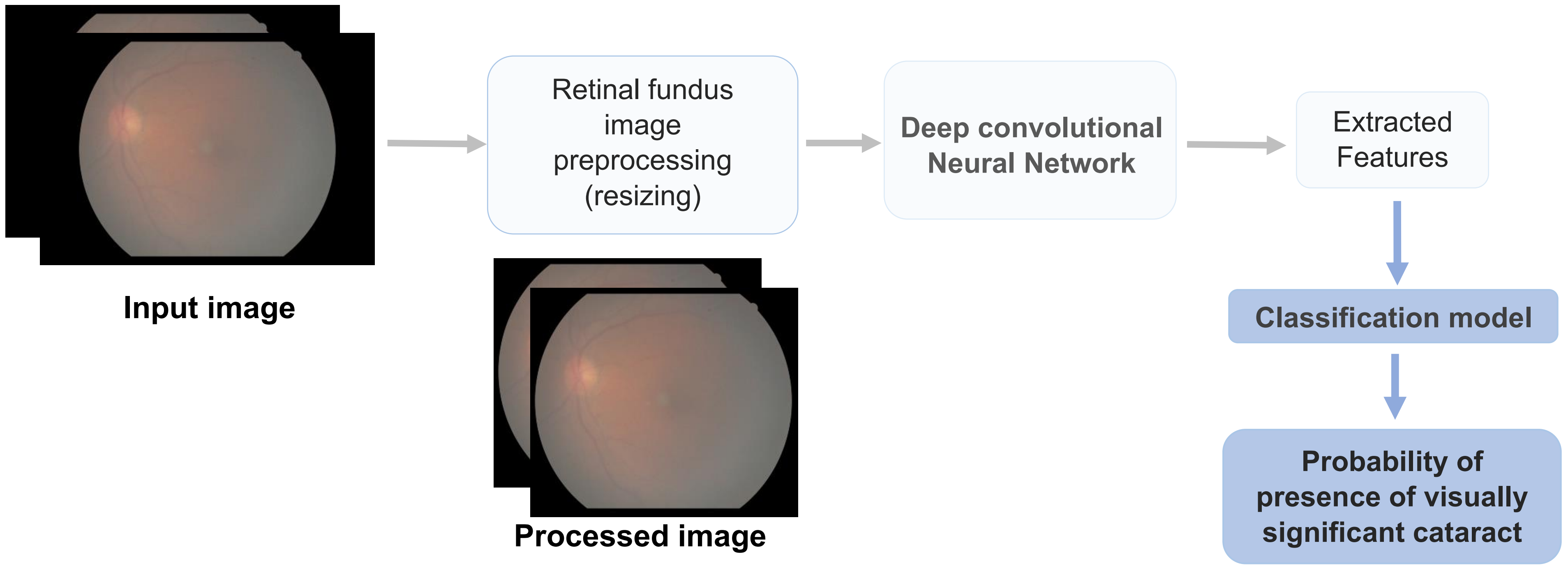

Supplementary Notes

Details on model development

In this study, we developed a deep learning model for the detection of visually significant cataract. We used macular-centered retinal images as the input to the deep learning model for the binary classification of visually significant cataract. The SIMES cohort dataset (n=5,038; 9,737 eyes) was randomly distributed into a development set (n=4,138), and an independent internal test set (n=900; 1,692 eyes) based on an 8:2 ratio at individual level (i.e. division done at person-level). As illustrated in **Annex Figure 1** below, the developed model pipeline consisted of two parts: a deep convolutional neural network (CNN), serving as a feature extractor, and the classification model.

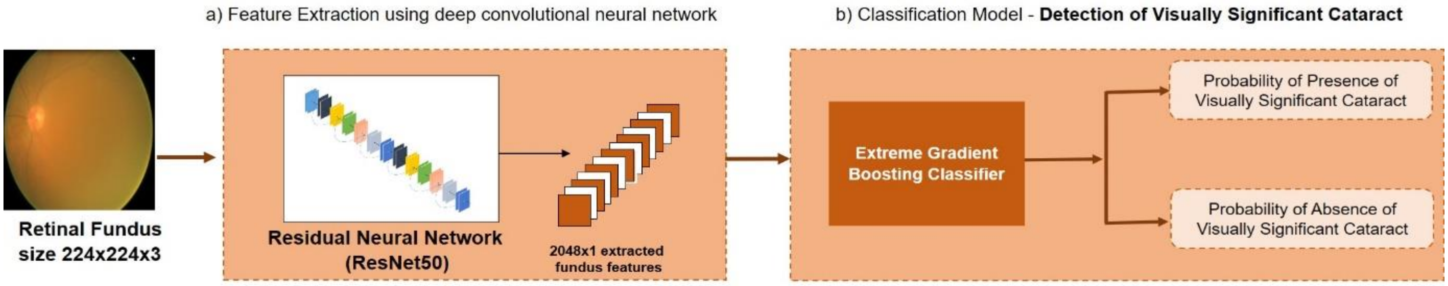

Annex Figure 1: The model pipeline.

The first step was feature extraction. For this purpose, a deep convolutional neural network called the Residual Neural Network (ResNet) was used.[1] A ResNet typically consists of multiple residual blocks, each of which contains a few convolutional layers and a residual connection to combine earlier features.[1] In particular, the network architecture used in our model was ResNet-50. Following the common practice for improved performance, we adopted a ResNet-50 model that was pre-trained on the ImageNet dataset.[2] The training retinal images were fed to the model to extract their features, a process referred to as 'feature extraction'. In this instance, 2,048 features were extracted from each training image. These features, along with the ground-truth clinical labels, were then used to classify the image through an extreme gradient boosting (XGBoost classifer) classification model.[2] The XGBoost classifer method was based on the gradient boosting approach, where decision trees were gradually added,

such that each subsequent tree reduced error of the preceding ones.[2] This method was aimed to prevent overfitting using the regularization techniques, parallelized tree building, tree pruning and other enhancement features.[2] The parameters for the XGBoost classifier, such as learning rate, minimum sum of instance weight needed, maximum depth of the tree, number of estimators, were chosen using the grid-search approach, in order to minimize its cross-validated classification error on the training set. In addition, since the dataset was imbalanced, we also adjusted the classifier parameters in order to balance the impact of positive and negative samples. Once the model had been trained, it was used for making predictions on the independent internal and external test dataset. The final output of the classification-based model was the probability for presence of visually significant cataract in each study eye.

Details on generating saliency maps

In order to understand which regions of the retinal images were used in the prediction decision for presence of visually significant cataract, we implemented the GradCAM method for generating saliency maps.[3] We replaced the gradient boosting module with a dense layer as the classifier. We first forward-propagated the images through the deep learning model to obtain predictions of the images. Once the prediction to the image was obtained, the gradients for the target class (i.e., the ground-truth class of the image) were set to 1, while the gradients for other classes were set to 0. The gradients were then backpropagated through the network. From a prespecified convolutional layer, the gradients and feature maps were extracted and combined to generate the heatmap (Gradient-based class activation map).[3] The saliency maps were overlaid on the original images to indicate the important regions.

References

1. He K, Zhang X, Ren S, Sun J. Deep residual learning for image recognition. Proceedings of the IEEE conference on computer vision and pattern recognition; 2016; 2016. p. 770-8.

2. Chen T, Guestrin C. Xgboost: A scalable tree boosting system. Proceedings of the 22nd acm sigkdd international conference on knowledge discovery and data mining; 2016; 2016. p. 785-94.

3. Selvaraju RR, Cogswell M, Das A, Vedantam R, Parikh D, Batra D. Grad-cam: Visual explanations from deep networks via gradient-based localization. Proceedings of the IEEE international conference on computer vision; 2017; 2017. p. 618-26.
