# Supplementary material for: Detecting visually significant cataract using retinal photograph-based deep learning
Source: Nat Aging. 2022 Feb 21;2(3):264–71. doi: 10.1038/s43587-022-00171-6 (PMC10154193; doi:10.1038/s43587-022-00171-6)
Supplement: Supplementary file 2 — Reporting Summary [file 43587_2022_171_MOESM2_ESM.pdf]

Corresponding author(s): Ching-Yu Cheng

Last updated by author(s): Jan 6, 2022

# Reporting Summary

Nature Research wishes to improve the reproducibility of the work that we publish. This form provides structure for consistency and transparency in reporting. For further information on Nature Research policies, see our Editorial Policies and the Editorial Policy Checklist.

## Statistics

For all statistical analyses, confirm that the following items are present in the figure legend, table legend, main text, or Methods section.

| n/a | Confirmed | |
|---|---|---|
| ☐ | ☒ | The exact sample size ($n$) for each experimental group/condition, given as a discrete number and unit of measurement |
| ☐ | ☒ | A statement on whether measurements were taken from distinct samples or whether the same sample was measured repeatedly |
| ☒ | ☐ | The statistical test(s) used AND whether they are one- or two-sided<br>*Only common tests should be described solely by name; describe more complex techniques in the Methods section.* |
| ☐ | ☒ | A description of all covariates tested |
| ☐ | ☒ | A description of any assumptions or corrections, such as tests of normality and adjustment for multiple comparisons |
| ☐ | ☒ | A full description of the statistical parameters including central tendency (e.g. means) or other basic estimates (e.g. regression coefficient) AND variation (e.g. standard deviation) or associated estimates of uncertainty (e.g. confidence intervals) |
| ☒ | ☐ | For null hypothesis testing, the test statistic (e.g. $F$, $t$, $r$) with confidence intervals, effect sizes, degrees of freedom and $P$ value noted<br>*Give P values as exact values whenever suitable.* |
| ☒ | ☐ | For Bayesian analysis, information on the choice of priors and Markov chain Monte Carlo settings |
| ☒ | ☐ | For hierarchical and complex designs, identification of the appropriate level for tests and full reporting of outcomes |
| ☒ | ☐ | Estimates of effect sizes (e.g. Cohen's $d$, Pearson's $r$), indicating how they were calculated |

*Our web collection on statistics for biologists contains articles on many of the points above.*

## Software and code

Policy information about availability of computer code

| | |
|---|---|
| Data collection | As the study was retrospective, no softwares were used for data collection purposes. Visually significant cataract was graded and defined based on the Wisconsin grading system and the Age-related Eye Diseases Study (AREDS) grading system. |
| Data analysis | We used TensorFlow (version 1.14.0) for development of the algorithms, including packages such as Torch (version 1.8.0), Torchvision (version 0.9.0), OpenCV (version 3.4.3.18), scikit-learn (version 0.20.02) and XGBoost (version 0.82).<br><br>The testing code used in this study can be accessed at https://doi.org/10.5281/zenodo.5650719. As the optimized algorithm is currently undergoing patent examination process, custom codes can be made available for research purpose from the corresponding author (Prof Ching-Yu Cheng) upon reasonable request. All requests for code will be reviewed by the SingHealth Intellectual Property Unit, to verify whether the request is subject to any IP or confidentiality constraints. Any code that can be shared will be released via a Material Transfer Agreement for non-commercial research purposes under the Creative Commons Attribution NonCommercial-NoDerivatives 4.0 license.<br><br>We performed the statistical analyses using standard statistical softwares (STATA, version 16, Texas; R version 1.1.456). |

For manuscripts utilizing custom algorithms or software that are central to the research but not yet described in published literature, software must be made available to editors and reviewers. We strongly encourage code deposition in a community repository (e.g. GitHub). See the Nature Research guidelines for submitting code & software for further information.

## Data

Policy information about availability of data

All manuscripts must include a data availability statement. This statement should provide the following information, where applicable:

- Accession codes, unique identifiers, or web links for publicly available datasets
- A list of figures that have associated raw data
- A description of any restrictions on data availability

The main data supporting the results in this study are available within the paper and its supplementary information. The retinal images and patient information are not publicly available due to patient privacy and the data is meant for research purposes only. On reasonable request, de-identified individual-participant data from the SIMES, SCES and SINDI datasets may be made available for academic purposes from the corresponding author (Prof Ching-Yu Cheng), subject to permission from the local institutional review board. Any data that can be shared will be released via a Material Transfer Agreement for non-commercial research purposes.

Data from the BES dataset cannot be readily released due to patient privacy and the data is meant for research use only. Reasonable requests for data from the BES cohort should be made directly to Professor Jost Jonas (email: jost.jonas@medma.uni-heidelberg.de) for consideration. Data can be made available for research purposes, subject to permission from the local institutional review board.

# Field-specific reporting

Please select the one below that is the best fit for your research. If you are not sure, read the appropriate sections before making your selection.

☒ Life sciences ☐ Behavioural & social sciences ☐ Ecological, evolutionary & environmental sciences

For a reference copy of the document with all sections, see nature.com/documents/nr-reporting-summary-flat.pdf

# Life sciences study design

All studies must disclose on these points even when the disclosure is negative.

| | |
|---|---|
| Sample size | Due to the nature of this study (outcome of interest is visually significant cataract), the relevant data needed for this study is rare and hard to be curated. Therefore, we included whichever datasets with the relevant data available and of large sample sizes (more than 5,000 eyes). No sample size calculation was performed. |
| Data exclusions | Across the development and test sets, study participants with incomplete or missing cataract grading or best-corrected visual acuity (BCVA) data, pseudophakic or aphakic eyes were excluded. Study eyes with visual impairment caused by other pathologies such as DR, age-related macular degeneration, and other maculopathy were excluded. In this study, only macula-centered retinal photographs were used. When multiple photographs were available for the same eye, only one photograph with the best quality was selected. Retinal photographs were further excluded from this study if the quality of the photographs was severely affected by artefacts due to eye movements, blinking, and insufficient illumination. |
| Replication | We successfully performed performance validation in three external validation datasets (the Singapore Chinese Eye Study, Singapore Indian Eye Study, Beijing Eye Study) |
| Randomization | Samples were randomly allocated to the training and validation datasets. |
| Blinding | Because the study was retrospective, no blinding was necessary. Splits for training and validation were random and automatically generated. |

# Reporting for specific materials, systems and methods

We require information from authors about some types of materials, experimental systems and methods used in many studies. Here, indicate whether each material, system or method listed is relevant to your study. If you are not sure if a list item applies to your research, read the appropriate section before selecting a response.

## Materials & experimental systems

| n/a | Involved in the study | |
|---|---|---|
| ☒ | ☐ | Antibodies |
| ☒ | ☐ | Eukaryotic cell lines |
| ☒ | ☐ | Palaeontology and archaeology |
| ☒ | ☐ | Animals and other organisms |
| ☐ | ☒ | Human research participants |
| ☒ | ☐ | Clinical data |
| ☒ | ☐ | Dual use research of concern |

## Methods

| n/a | Involved in the study | |
|---|---|---|
| ☒ | ☐ | ChIP-seq |
| ☒ | ☐ | Flow cytometry |
| ☒ | ☐ | MRI-based neuroimaging |

## Human research participants

Policy information about studies involving human research participants

| | |
|---|---|
| Population characteristics | Individuals aged 40 and above, and with relevant clinical data available (as described above) were included for this evaluation. |
| Recruitment | The included datasets were all population-based studies with random sampling performed during recruitment. Participants were given reimbursement for their time and effort. |
| Ethics oversight | All included studies adhered to the tenets of the Declaration of Helsinki and had respective local ethical committee approval. We obtained permission from the principal investigator of each study to use the data. |

Note that full information on the approval of the study protocol must also be provided in the manuscript.
